# Protection for the Bodies of the Dead

**Published:** 1886-12

**Authors:** 


### Editorial.

### PROTECTION FOR THE BODIES OF THE DEAD.

The *Christian Index*, of November 18th, edited by Rev. H. H. Tucker, D. D., one of the ablest men in Georgia, contains a timely editorial on this subject, which so nearly meets our views that we reproduce it in full without comment, except to say that if the Legislature longer delays making necessary provision for the medical colleges, they thereby place a premium upon crime.

"There is before the General Assembly a bill for the protection of cemeteries, which we hope will pass and become a law. There are several medical colleges in the State. These must have, and ought to have, and *will* have human bodies for anatomical purposes. The question is, where and how shall they get these bodies ? At present there is no lawful way of getting them; hence, of course, they must and *will* be had, and *are* had, *un*lawfully. There is absolutely no protection for our dead. Any cemetery, public or private, is liable to be raided on and robbed. In the cities there is some little protection possibly by the police, whose vigilance might discover any such desecration of the place of the dead, but in the country, where there is no police, any grave is liable to be torn open and violated. The body of one dear to family and kindred at least is liable to be exposed to the gaze of students and cut to pieces. The only way to prevent this shocking horror is to provide means whereby the medical colleges can be supplied *lawfully*.

"The bill now before the Legislature provides that bodies, which are to be buried at public expense, and which are *unclaimed* by any relative or friend for three months, shall be used for anatomical purposes, provided that the remains, after dissection, shall be decently buried. Ample material can be supplied to the colleges in this way, and no grave will be robbed, and nobody's feelings will be hurt, and no one need feel uneasy as to the graves of his kindred or as to his own. This is the only way of stopping the horrid work of grave-robbing, and we hope that the Legislature will pass the bill."

